# Mediterranean Diet: The Role of Phenolic Compounds from Aromatic Plant Foods

**DOI:** 10.3390/foods12040840

**Published:** 2023-02-16

**Authors:** Amélia Delgado, Sandra Gonçalves, Anabela Romano

**Affiliations:** 1 MED—Mediterranean Institute for Agriculture, Environment and Development & CHANGE—Global Change and Sustainability Institute, Universidade do Algarve, 8005-139 Faro, Portugal; 2 Faculdade de Ciências e Tecnologia, Universidade do Algarve, Campus de Gambelas, Ed. 8, 8005-139 Faro, Portugal

**Keywords:** Mediterranean Diet, plant foods, bioactive compounds, culinary herbs, biodiversity loss, plant species’ preservation

## Abstract

Today’s global food system aggravates climate change while failing in meeting SDG2 and more. Yet, some sustainable food cultures, such as the Mediterranean Diet (MD), are simultaneously safe, healthy, and rooted in biodiversity. Their wide range of fruits, herbs, and vegetables convey many bioactive compounds, often associated with colour, texture, and aroma. Phenolic compounds are largely responsible for such features of MD’s foods. These plant secondary metabolites all share in vitro bioactivities (e.g., antioxidants), and some are evidenced in vivo (e.g., plant sterols lower cholesterol levels in blood). The present work examines the role of polyphenols in the MD, with respect to human and planetary health. Since the commercial interest in polyphenols is increasing, a strategy for the sustainable exploitation of Mediterranean plants is essential in preserving species at risk while valuing local cultivars (e.g., through the geographical indication mechanism). Finally, the linkage of food habits with cultural landscapes, a cornerstone of the MD, should enable awareness-raising about seasonality, endemism, and other natural constraints to ensure the sustainable exploitation of Mediterranean plants.

## 1. Introduction

The Mediterranean basin is among the richest and most complex regions on Earth: geologically, biologically, and culturally. It is a living moving mosaic because the natural landscapes and climate shaped civilizations, and we have in turn shaped nature, in a kind of symbiotic relationship, allowing a high degree of endemism species as well as the adaptation of introduced ones [1]. The Mediterranean peoples have learned to cope with nature and in building resilience to thrive. The same applies to nature in regenerating and adapting. However, the combined stresses faced by the Mediterranean region today are unprecedented and this region has been considered a climate impact hotspot and a global priority place by important international organizations [2,3].

Food production (from farm to fork) plays a key role with respect to Mediterranean natural assets, either by enhancing their conservation or their depletion. Industrialization and globalization brought mass production, overconsumption, different food habits with associated public health burdens, freshwater scarcity, biodiversity loss, and more. However, the combination of ancient wisdom with innovation and awareness-raising has been advocated in ensuring sustainable development and conservation of resources [1].

According to the United Nations Food and Agriculture Organization (FAO), a sustainable food system is such that ensures a sufficient supply of safe and nutritious food for all, ensuring economic sustainability (by being profitable throughout the value chain), social sustainability (by bringing wellness to local communities and preserving their cultural assets), and environmental sustainability (because of its nature-regenerative character or neutral impact on the planet) [4]. However, the mainstream global food systems have been pointed out as failing in all these sustainability pilasters, mainly because only short-term profits are sought, and food commodities are produced at the cost of low wages and environmental degradation. Food production is a driver of the devastation of natural capital, including massive biodiversity loss, soil degradation, and more [5,6]. There is, however, hope for fixing food systems, since in some regions of the world healthy and sustainable food habits can still be found [7], as is the case of the Mediterranean Diet (MD), which is a cultural asset recognized by the United Nations Educational, Scientific, and Cultural Organization (UNESCO) as an intangible heritage of the humankind [8]. Yet, MD is best known for its food pattern, which is well-known for being simultaneously healthy and sustainable [1,9,10].

Since the MD is mainly plant-based, we focus the current review on the use of Mediterranean culinary herbs, revealing how impacts on human and planetary health are connected (one health), meaning that adequate food choices may strongly contribute to improving public health and nutrition while minimising the environmental footprint of foods and valorising biodiversity. The importance of spices and culinary herbs in Mediterranean cuisine is first associated with the colours, aromas, and textures conveyed to the dishes, notably by phenolic compounds. Their main classes and chemical properties are summarised as well as their occurrence in spices and culinary herbs and associated health-promoting effects. Finally, we highlight some threatened (edible) aromatic plants in the Mediterranean, and we discuss ways of sustainably exploiting and protecting them. Having in mind these constraints, improving the adherence scores to the MD is expected to simultaneously address current public health issues, adapt to climate change, and restore more sustainable and resilient food systems.

## 2. A Broken Global Food System. What Way Out?

It is well acknowledged that the way we currently produce, transform, distribute, and consume food exceeds planetary boundaries. Bold strategies with time-bound objectives have been set aiming to reverse this trend, such as Farm to Fork and the European climate law from the European Commission [11] and the Sustainable Development Goals (SGDs) proposed by the United Nations [12].

When it comes to food, the complexity of the issues involved has to be acknowledged, because food is much more than a sum of nutrients, and food choices involve from primary senses to memories, beliefs, and more [13,14,15,16,17,18], as is the case of the MD.

Conversely, mainstream globally available foods result from the obtainment of macronutrients (e.g., protein, fat) from monocrops factory farms by extracting and modifying food components into food ingredients, which are later further processed and blended with synthetic compounds in formulating ultra-processed foods (UPF) [19,20].

This way of producing and consuming food has been causing natural capital losses, such as agro-biodiversity depletion (because of the preference for monocrops) and environmental damage (caused by pollution and deforestation), in addition to public health issues (notably the double burden of obesity and malnutrition) [20,21,22,23], not to mention the contribution to the erosion of sustainable and healthy food cultures, such as the MD [1]. Despite the acknowledged multi-level damage, the consumption of UPF continues to be supported by strong marketing campaigns largely targeting children [24,25,26,27], most probably contributing to the lower and lower adherence scores to the MD, which have been noted especially among youngsters [28,29,30,31].

Conversely, high adherence scores to the MD can significantly enhance the nutrition quality and public health of populations [10,32,33,34,35] while being respectful of the environment [36,37,38] and, in certain cases, contributing to the preservation of agro-biodiversity [39,40]. A change in the food system paradigm has been proposed by many, mostly consisting in promoting the adoption of more sustainable diets, with many similarities to the MD (e.g., planetary diet by the EAT-Lancet commission).

## 3. The Mediterranean Diet Is Simultaneously Healthy, Sustainable, and Rooted in Biodiversity

Bach-Faig and colleagues [41] described the MD as a “dietary pattern and associated lifestyle that adopts mainly plant foods. It is rich in fruits, vegetables, bread, pasta, rice, couscous, and other cereals, as well as olives, nuts, seeds, herbs, spices, garlic, onions, legumes, potatoes, and more. Olive oil is the main fat, and ‘biodiversity’ and seasonality define this dietary pattern, which embraces a wide variety of plant foods of local origin and their seasonal character”. When speaking about Mediterranean cuisine, the still existing biodiversity translates into a wide assortment of textures, flavours, and colours of the dishes, varying from one place to the other, according to preferences and availabilities. It can be said that many MD exists because of regional differences, but in fact, these result from making the best use of local resources and embracing biodiversity [1].

**Figure 1:**
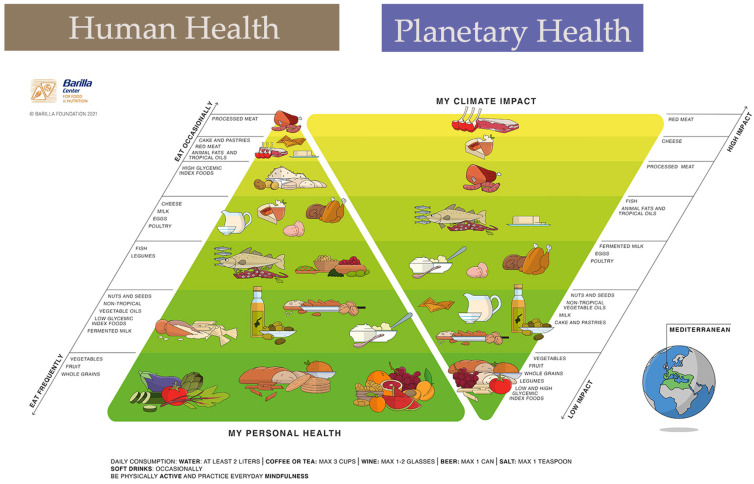
One-health outcomes of the Mediterranean Diet according to the double pyramid model (Source: Barilla Foundation 2021 [7]).

The way human and planet health (pollution, biodiversity conservation) are aligned in sustainable diets is shown in Figure 1, which refers to the MD. The double pyramid model is based on composite indicators that encompass health outcomes and environmental impacts of foods and is described in [7,9,37,42]. It is organized proportionally to the expected impact, with the larger area of the pyramid corresponding to the highest impact and vice versa.

Therefore, the pyramid shown on the left side of Figure 1 corresponds to the MD’s dietary recommendations and shows that the foods, from the bottom of the pyramid, when consumed in larger amounts and more frequently correspond to more positive health outcomes with reduced environmental impacts. As can be observed in Figure 1, plant foods are found at the bottom row and include fruits and culinary herbs. MD meals bring biodiversity into the plate, consisting of a balanced blend of colours (mainly conveyed by plant pigments), textures (because the food matrix is not destroyed, as in UPF), and complex aromas (mostly from aldehydes and phenolic compounds).

## 4. Role of Aromatic Plants in the MD Cuisine

Culinary traditions between Mediterranean countries changed significantly over the past decades, but the habit of enriching food with flavours and aromas remains across the Mediterranean countries [41,43]. Aromatic herbs are essential ingredients of MD, used as food additives and condiments, and as herbal teas [44]. Aromatic herbs can be a pleasant and healthier substitute for salt (NaCl) in cooking. Oregano (*Origanum vulgare* L.), thyme (*Thymus vulgaris* L.), sage (*Salvia officinalis* L.), and rosemary (*Rosmarinus officinalis* L.) are well-known aromatic herbs, which belong to the Lamiaceae family. On the other hand, the mints (a large number of species and subspecies) are widely used as medicinal aids (in folk medicine) because of their reported health-promoting actions on top of their aromas. Lemon balm (*Melissa officinalis* L.), Peppermint (*Mentha piperita* L.) and kitchen mint (*Mentha spicata* L.) are examples of species with subspecies found only in specific habitats.

The richness of these herbs in bioactive compounds has been attracting the attention of the pharma and food industries in meeting consumers’ demand for innovative products of “natural origin”, from cosmetics to food supplements [45]. This market demand may turn the spotlights onto some species, valuing them. However, it should be ensured that regulations for sustainable exploitation are integrated with biodiversity conservation strategies.

The Mediterranean basin (still a hotspot of biodiversity) harbours many endemic species including aromatic herbs, such as *Origanum dictamnus* L. (known as dittany) endemic of Crete Island, *Thymus lotocephalus* G. López & R. Morales, found in Algarve, Portugal, or the *Capsicum annuum* cv holding the protected designation of origin (PDO) “pemento de Herbón”, commonly known as pimiento de Padrón, only found in a certain region of Galicia, Spain [46].

## 5. Phenolic Compounds

Mediterranean aromatic plants are generally rich in phenolic compounds (also known as phenols or phenolics). This designation refers to a broad range of bioactive molecules sharing the below-described features. They can be found in variable amounts in plant foods and are involved in colour (e.g., pigments), flavour (e.g., responsible for astringency, bitterness), and food safety (due to antimicrobial activity). Phenolic compounds from foods are most often valued for their general antioxidant character and they have been recently categorized as phytonutrients because of the mounting evidence and growing awareness of their health-promoting features [47,48,49]. Phenolic compounds exist in fresh vegetables (e.g., leafy vegetables, aromatic herbs, nuts) and processed (fruit juice, tea, coffee, wine) and, although their release kinetics during food digestion and bioavailability afterwards are still unclear, they seem to depend on the food matrix, on the interactions with other nutrients and more [50,51,52].

From a botanical viewpoint, phenolic compounds are ubiquitous in the Plant Kingdom. They are mainly plant secondary metabolites involved in plant morphology, reproduction, growth, resistance against predators and pathogens, and more [53]. The concentration of a certain phenolic compound may vary from organ to organ within the same plant, as does the distribution of compounds (phenolic profile). For example, Gorzynik-Debicka et al. [54] noted variations from 50 to 1000 mg/kg.

**Figure 2:**
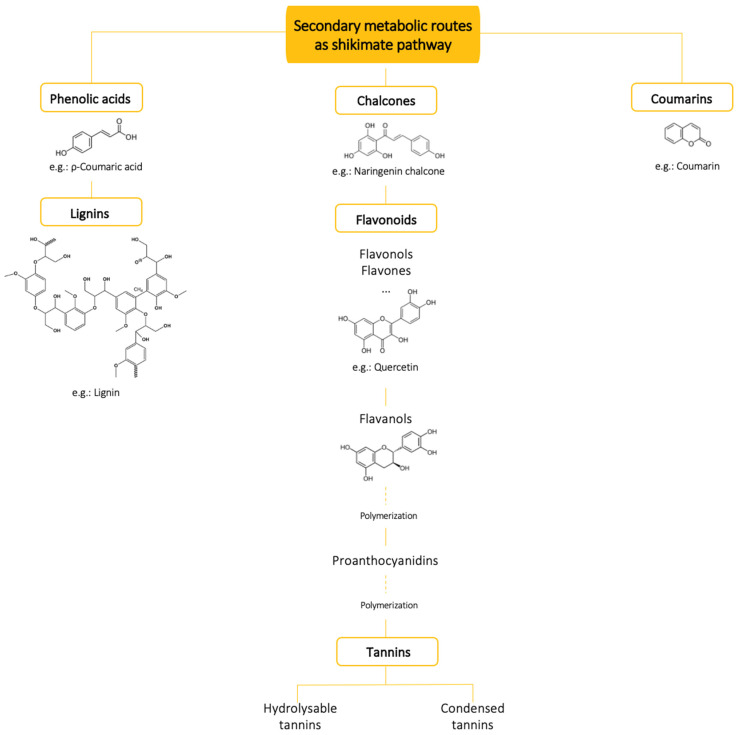
Main groups of phenolic compounds in Mediterranean aromatic plants.

Figure 2 summarises the main groups of plant phenolic compounds also found in Mediterranean aromatic herbs. Some examples are included and more information (e.g., culinary uses, bioactivities) can be found in Table 1.

### 5.1. Categorizing Phenolic Compounds from Plant Foods

Phenolic compounds are a heterogeneous group of plant secondary metabolites, having in common the presence of at least one aromatic ring and hydroxyl group (OH), which is the root for their name, as phenol is the simplest compound with such features. The designation “phenolic compounds” is broad enough to include from simple molecules to polymers, diverging not only in size but also in chemical properties (as polarity) due to differences in functional groups (such as ester and acid). Given the heterogeneity of this group of compounds, they have been named and categorized differently by different authors according to several viewpoints [55].

When categorizing phenolics regarding plant metabolic pathways, three large groups can be first considered: phenolic acids, chalcones, and coumarins (Figure 2) because they can be precursors of others [55]. Phenolic acids can be precursors in the synthesis of lignins. In another pathway, chalcones may originate flavonoids, which, in turn, may condensate. Polymerization proceeds; proanthocyanidins are first oligomers and then polymers composed of units of flavanols. These polymers may ultimately originate tannins, of high molecular mass. Based on different properties, two subclasses can be considered, hydrolysable and condensed tannins [56]. Coumarins, the third large group above-referred, do not undergo any further transformation [57].

Because plant phenols are secondary metabolites, it means that they come from one of the myriad existing ramifications of metabolic pathways (secondary pathways), including some that are poorly understood. Such complexity explains why certain phenols are genus-specific or species-specific while others seem to be more widely distributed. It should be noted that each plant food has a particular phenolic profile, which is rich and complex with the Mediterranean aromatic herbs, as disclosed below. The correlation with health outcomes in the context of the MD, is also inspected, namely in 6.

When categorizing phenolics regarding their chemical structure, as for the number of aromatic rings and the number of carbon atoms, they can be divided into two main classes, further detailed below: simple phenols and polyphenols, which are of a more complex structure. Examples of simple phenols are hydroxybenzoic acid and hydroxycinnamic acid (and derivatives). Examples of polyphenols are lignans, flavonoids, and tannins [55].

When considering overall function, structure, and occurrence, some types of compounds stand out.

#### 5.1.1. Simple Phenols and Derivatives

Firstly, simple phenols notably encompass hydroxycinnamic acid and derivatives and hydroxybenzoic acids and derivatives [55]. 4-Hydroxycinnamic acid (also called p-coumaric acid) plays an important role in plant metabolism and contributes to the flavour of plant foods (Figure 2). The molecule is non-polar, and the substance is solid under normal environmental conditions. Associated health-promoting properties include anti-microbial and free-radical scavenging actions. Caffeic acid and cinnamic acid are related compounds often found in plant foods [58]. Also important in plant metabolism is 4-hydroxybenzoic acid. Moreover, it is a worldwide authorized food additive (as is benzoic acid). Benzoic acid, picolinic acid, and gallic acid are related compounds [58,59].

Lignans are dimers of phenylpropanoid units linked by the central carbons of their side chains [56] and derivate from phenolic acids [57]. It is noteworthy that dibenzylbutane derivatives, occurring in higher plants, can be synthesized by human gut microbiota in vitro [58]. Some plant lignans are phytoestrogens, which are estrogen-like molecules that resemble the human hormone. Phytoestrogens can be metabolized by intestinal bacteria, indirectly interfering with human metabolism. Other lignans can also be metabolized in the gut, most probably playing relevant roles in human health and wellbeing. These include pinoresinol, lariciresinol, secoisolariciresinol, matairesinol, hydroxymatairesinol, syringaresinol, and sesamin [59]. Lignins (Figure 2) are polymers of lignan units and act as antioxidants as practically all other polyphenols.

#### 5.1.2. Chalcones and Derivatives

Chalcones are aromatic ketones, which can be precursors of other polyphenols, notably flavonoids. Associated health-promoting properties include anti-microbial, anti-tumour, and anti-inflammatory actions [59].

In its turn, as roughly overviewed in Figure 2, flavonoids can be subdivided into flavonols, dihydroflavonols, isoflavones, and flavanols, according to the degree of hydrogenation and hydroxylation of their three-ring structure [56,58]. Flavonoids bind easily to sugars resulting in a pigment whose colour grade depends on the nature of the chemical bond established between the phenol moiety and the sugar residue [57].

Flavonols have the 3-hydroxyflavone backbone in common, but the position of the hydroxyl (OH) group may vary. Flavonol aglycones, in living plants, act as potent antioxidants, being part of a protective mechanism against damage from reactive oxygen species. Kaempferol and quercetin belong to this group. They are quite ubiquitous and have been attracting more and more attention, as noted by Issaoui et al. [60].

Quercetin is ubiquitous in plant food sources (Figure 2, Table 1). It is a potent antioxidant with potential chemo-preventive and anti-inflammatory activities. The glycoside form has a higher bioavailability and, when metabolised in the gut, will probably have beneficial effects on the intestinal mucosal epithelium [61]. It is believed that quercetin glycosides are converted into phenolic acids as they travel along the colon [59]. Similarly, kaempferol is thought to enhance the intestinal barrier by acting at the level of epithelial cell tight junctions, and it has also been noted that kaempferol may contribute to the prevention of obesity and diabetes [61]. Because of its strong capacity to reduce oxidative stress, kaempferol has been proposed as an adjuvant in cancer treatment [58,59].

Onion is probably the richest food source of kaempferol and quercetin [62], but oregano may also be a relevant dietary source of quercetin [59,61].

Flavanols, also referred to as flavan-3-ols or catechins (Figure 2), are derivatives of flavans and include catechin, epicatechin gallate, epigallocatechin, epigallocatechin gallate, proanthocyanidins, and thearubigins [59]. According to the same authors, catechin is water soluble and can chelate heavy metals and bind to proteins, including bacterial toxins of proteinaceous nature, which may explain its antimicrobial and detoxifying properties. Catechin is mostly found in green tea but is also present in rosemary.

Catechin and epigallocatechin may polymerize together in different proportions, and the levels of antioxidant activity seem to depend on the degree of polymerization (Figure 2). Condensed tannins are obtained after a series of condensation and polymerization reactions [57,63].

Condensed tannins are thus flavanol polymers not readily hydrolysed that are responsible for the astringency taste of many plant foods [57]. As polyphenols in general, they were regarded by nutritionists as antinutrients, to be avoided. However, state-of-the-art knowledge, especially about the human microbiome, has been disclosing their relevance as phytonutrients [64]. According to Selma et al. [65], dietary polyphenols act as phytonutrients by first interacting with the gut microbiota and often being transformed by bacteria before entering the bloodstream. Still, according to the same researchers, some health benefits from phenolic compounds may result, to a large extent, from such microbial bioactive metabolites.

#### 5.1.3. Coumarins

Thirdly, and finally, the designation “coumarins” includes an array of benzo-alpha-pyrone compounds with important and diverse physiological activities in living plants as well as in human metabolism. The parent compound, coumarin (Figure 2), occurs often in spices (e.g., cinnamon), aromatic herbs (such as peppermint), and in honey from lavender. Coumarin is an antineoplastic agent also used to treat venous insufficiency [59].

## 6. Phenolic Compounds in Mediterranean Aromatic Plants

The Mediterranean cuisine is simple but rich, and the abundant use of seasonings (herbs and spices, and a few plain ingredients) is noteworthy. Aromatic herbs and spices convey colours and flavours varying from season to season and from region to region in a balanced way. Many of these herbs are commonly grown as kitchen-garden herbs, and while some are perennial and evergreen (such as rosemary and mints), others have a more seasonal character, such as coriander and basil. Aromatic herbs used in Mediterranean cuisine are rich in bioactive compounds and, thus, contribute to the MD’s health benefits. Some documented health outcomes of spices and herbs used in Mediterranean cuisine are highlighted in Table 1, which is focused on the native plants of the region. Many more herbs could be added to the list as well as traditionally used spices, from the orient, such as cinnamon and cloves, but those were intentionally not included.

**Table 1:** Prominent phenolic compounds, health-promoting actions and other properties of aromatic herbs commonly used in Mediterranean cuisine.

Common Name/Species	Culinary and Folk Medicine Uses	Reported Phenolic Compounds	Evidence-Based Health Outcomes	References
Coriander/*Coriandrum sativum*	Both, the fresh leaves, and seeds can be used as a seasoning, with fresh leaves generally conveying a more intense aroma to rice dishes, salads, stews and more;	The flavonol quercetin ^1^, is reported along with gallic, protocatecuic and ferulic acids;	Antioxidant, anti-cancer, anti-microbial, anti-thrombogenic, and neuroprotective;	[45,62,66]
Oregano/*Origanum vulgare* L.	Very popular seasoning for salads and pizza; in folk medicine, it is believed to act as an appetiser, diuretic and anti-flatulence;	Wide range of simple phenols and phenolic acids such as thymol, carvacrol, rosmarinic acid, as well as flavonoids as naringenin, apigenin ^1^, luteolin ^1^, quercetin ^1^ and tannins;	Antioxidant, antimicrobial, immunomodulatory, anticancer;	[45,66,67,68]
Thyme/*Thymus vulgaris*	Almost mandatory in pesto (a well-known Italian sauce); folk medicine prescribes it to tackle infection and inflammation of the respiratory tract;	Besides the flagship compound, thymol, thyme is rich in flavonoids such as apigenin ^1^ and luteolin ^1^; other simple phenols as carvacrol, phenolic acids as rosmaniric and caffeic acids, have also been reported;	Antioxidant, anti-bacterial and anti-fungic activities, prevent atherosclerosis and seems to have some anti-neoplastic action;	[45,66]
Rosemary/*Rosmarinus officinalis* (syn. *Salvia rosmarinus*)	Widely used in the Mediterranean and other cuisines to season roasted meats, appetizers, and more; it has been used as food preservative;	Besides the flagship compound, rosmaniric acid, also reported are caffeic and carnosic acids, carnosol and rosmanol, in addition to the flavonoids naringin and apigenin ^1^;	Antioxidant, neuroprotective and anti-neoplastic activities; it is also referred to lower blood lipid’s level;	[45,62,69,70,71]
Peppermint (*Mentha piperita*)	The mint group comprises more than 60 species of different aromas, and all rich in phenolic compounds. They are popular kitchen garden herbs with many curative properties, according to folk medicine;	Menthol, catechin ^2^, cathechin-3-O-Gallate ^2^; epigallocatechin ^2^;	Antioxidant, antimicrobial, anti-inflammatory and local analgesic actions;	[62,72,73,74]
Basil (*Ocimum basilicum*)	The biodiversity within the “basil” group has been economically valorised by marketing varieties with different colours and aromas. Basil is widely used in pasta and salads;	Eugenol and a wide range of other phenolic compounds not identified and/or typical of certain cvs;	Antioxidant, anti-microbial, and anti-neoplastic activities;	[75,76]
Phennel (*Foeniculum vulgare*)	The whole plant can be used in culinary preparations, with meats, in stews, deserts or liquors;	Besides the flagship compound, p-Anisic acid, other reported phenolic acids are hydroxybenzoic, ferrulic and o- and p-coumaric acids, as well as the flavonoids isorhamnetin (related to quercetin), quercetin ^1^, myricetin ^1^, kaempferol ^1^, and luteolin ^1^;	Antioxidant, anti-inflammatory, and anti-diabetic properties;	[62,77,78]
Chilli-pepper (*Capsicum annuum*)	The fruits are used directly or smashed for a hot seasoning; Capsaicin is the pungent compound and the main bioactive molecule.	(Capsaicin); luteolin ^1^ and quercetin ^1^.	Antioxidant, analgesic, anti-cancer and anti-inflammatory properties.	[62,79,80]

Since phenolic compounds are secondary metabolites from plants, their occurrence and concentration levels can be highly variable and influenced by many factors such as water stress, pest attacks, and more. The presented list may not be exhaustive, and the enumerated compounds may be absent, from plants, under certain conditions. ^1^ The flavonoids luteolin, apigenin, quercetin, myricetin, kaempferol and gallic acid are absorbed during digestion and their metabolites have been identified and often quantified. Further information is searchable in the Human Metabolome Database (HMDB) [https://hmdb.ca/metabolites/ (accessed on 20 December 2022)]. ^2^ Awareness of the relevance of large molecules to human health, such as catechins and tannins, is increasing. Such compounds have been detected and quantified in the human body and information on their metabolites, enzymes and pathways can be retrieved from the Human Metabolome Database (HMDB) [https://hmdb.ca/metabolites/ (accessed on 20 December 2022)].

As can be observed in Table 1, the ability to neutralize free radicals and the antimicrobial character are common features of phenolic compounds, which can easily be proven in vitro. Studies on the mechanism of action of certain phenolics are also referenced but the number of published clinical studies is reduced.

Despite the popularity of herbal infusions in folk medicine and herbal extracts in alternative therapies, the scientific opinions issued by the European Food Safety Agency (EFSA) seldom support such claims. The main reasons are due to the wording that does not sufficiently detail the claim impairing a correct assessment of the cause–effect relationships (e.g., the claim “anti-inflammatory action” would correspond to the reduction of inflammation markers, which beneficial physiological effect depends on the particular context) [64]. Systematic reviews of clinical trials, as is the case of [81], also show little or inconclusive evidence of the benefits of herbs in the treatment of ailments.

Recent evidence on the modulation of gut microbiota by aromatic herbs [82,83,84] suggests relevant physiological effects are hand-in-hand with the complex flavours (notably bitterness notes) conveyed by herbs used in seasoning dishes.

Beneficial health outcomes are rarely attributed to a single compound but rather to whole foods and notably to the Mediterranean dietary pattern (MD), in which aromatic herbs play a key role. Mounting results of clinical studies support various health benefits of the MD (as referred above) instead of a few “superfoods”. The complex composition of foods, unknown interactions between the different compounds within the food matrix, and the changes they undergo during processing (which includes cooking) need to be considered. The role of the food matrix in the bioavailability of phytonutrients and other bioactive compounds from foods was stressed by [19].

## 7. Wild Mediterranean Aromatic Plants and Conservation Concerns

The Mediterranean Basin comprises high mountains, islands, and coastal areas that are remarkably rich in terms of biodiversity. High plant diversity and endemism characterise the area, which is considered the second largest terrestrial biodiversity hotspot in the world [85]. The habit of collecting wild edible plants for own consumption, namely to use as culinary herbs and in herbal teas, is a tradition of Mediterranean culture and an integral part of the Mediterranean diet [43]. Many Mediterranean herbs are narrowly distributed, or local endemics and their scent and flavour are potentiated by Mediterranean climatic conditions. Due to the presence of bioactive compounds, Mediterranean herbs provide not only an agreeable aroma and taste to food, but also improve food preservation while providing multiple health benefits [78]. It should be noted that plants, notably wild herbs, may contain health-promoting and/or poisoning compounds. Seldom, the same compound can be beneficial or deleterious depending on the dosage. The Mediterranean culinary herbs herein described have been used for centuries mainly as seasonings and may therefore be considered as GRAS (generally recognised as safe). Many locals have the necessary empirical knowledge to select the desired species in the wild including for use in folk medicine.

Many aromatic herbs are locally harvested to be used as basic ingredients in several dishes. However, some Mediterranean Lamiaceae herbs with potential relevance for the Mediterranean Diet are listed in the IUCN Red List of Threatened Species as “near threatened”, “vulnerable”, “endangered”, or “critically endangered” (Table 2). Examples can be found in some Mediterranean countries, e.g., *Origanum compactum* is classified as “vulnerable” in Spain; *O. vulgare* as “endangered” in Albania; several *Thymus* species, endemic from Portugal, are classified as “vulnerable” or ”near threatened”; and the mountain tea species *Sideritis euboea* and *S. sipylea* are classified as “endangered” in Greece [86,87]. Moreover, four plant species native to Crete (Greece) occurred in restricted populations and need conservation measures: *Origanum vulgare* subsp. *Hirtum* (Link) Ietsw. (oregano), *Salvia fruticosa* Mill. (sage), *Sideritis syriaca* L. subsp. *Syriaca* (malotira, a local mountain tea), and *Origanum microphyllum* (Benth.) Vogel (Cretan marjoram) [88].

The causes contributing to decline or extinction of plant species are mainly uncontrolled overcollection, urban and tourism pressure, and climate change. As noted before [2,3] and according to most probable scenarios from climate change models, the Mediterranean basin is one of the regions across the globe that will be expected to be strongly affected [89], negatively impacting crop quality and productivity. Environmental changes are of particular interest for the biosynthesis of secondary metabolites like phenolic compounds, namely in Mediterranean aromatic plants [90,91], consequently changing their corresponding health benefits. Despite such potential issues, we note a knowledge gap with respect to plant metabolic adaptation to climate change, since there are limited studies focusing on the impact of climate change on aromatic plants to the present date.

## 8. Concluding Remarks and Prospects

The linear economic view of food systems has been acknowledged, notably in COP summits, as detached from the pace of nature and from human physiological and cultural needs. Treating food as a commodity has increased disruption risk in food chains and has been contributing to the existence of food deserts (areas where people have no access to physical food markets), to food loss, and to waste. The mainstream way of regarding food production and consumption is out of planetary boundaries. The urge for a change is consensual among experts from different areas of knowledge because of the serious damage to human health (high prevalence of non-communicable diseases), the environment (pollution and biodiversity loss), cultural assets (threatening world heritage, when MD adherence scores are decreasing), and to the economy (on one hand because a few multinationals rule the current food systems, affecting regional sustainable development; on the other hand, because the wrong food choices reflect in growing expense with public health).

The lack of compliance with every sustainability pilaster is translated in the failure of current food systems to meet several SDGs and cope with climate change. The Mediterranean food culture, which is deeply connected to the territories, is likely to be directly and indirectly affected by the changing environmental conditions in the area, since in the Mediterranean basin, observations from the past decades and most probable scenarios from climatic models prescribe a drift towards a semi-arid climate. Anthropogenic pressure and extreme weather events are challenging the resilience of Mediterranean plants and driving many of them to extinction. Of particular concern is the increasing demand for wild aromatic species and their overharvesting together with climate change acceleration threatens the survival of wild populations of some aromatic species with a great role in the Mediterranean Diet. In practice, stimulating higher adherence levels to the MD includes raising awareness on the complex aromas and health benefits conveyed by phenolic compounds from local aromatic herbs, along with the associated environmental constraints, in the “one health” viewpoint (please see Section 6 and Section 7). Revamping the MD will certainly unlock knowledge-based applications, making use of the bioactivity of phenols with a consequent rising demand for aromatic herbs. However, having in mind that some species are already threatened, strategies for their sustainable exploitation urge. Therefore, restricting wild harvesting, stimulating sustainable cultivation, and applying conservation strategies are important to preserve local plant resources and natural heritage. These actions should be a joint effort of government entities, non-profit organizations, academia, local communities, and industrial companies as part of the effort in revamping sustainable and resilient food systems adequate to face increased risks of food insecurity and natural capital and economic losses.

Possible strategies for sustainable exploitation of Mediterranean plants may include the commercial valuation of local cultivars, which will benefit small local businesses. Tools to implement such strategy include promoting geographical indication seals, responsible marketing, and consumer literacy on healthy sustainable food habits as well as on nature’s preservation. Wild biodiversity and agro-biodiversity are as connected as are human and planetary health. Awareness-raising actions and citizen science about endemism and other natural constraints may trigger bottom-up pressure towards species conservation and more sustainable agricultural practices. In addition, awareness-raising and other local actions about the seasonal character of many foods and their health-related value will certainly highlight the linkage of food habits with cultural landscapes, a cornerstone of the MD, thus enabling the sustainable exploitation of Mediterranean plants.

## Figures and Tables

**Table 2:** Examples of Mediterranean aromatic species listed in the International Union for Conservation of Nature, IUCN Red List of Threatened Species.

Genus/Species	IUCN Red List Category	Geographic Range
*Mentha*		
*M. cervine*	Near Threatened	Algeria; France (mainland); Morocco; Portugal (mainland); Spain (mainland) Possibly Extinct in Italy (mainland)
*M. gattefossei*	Vulnerable	Morocco
*Origanum*		
*O. cordifolium*	Vulnerable	Cyprus
*O. dictamnus*	Near Threatened	Greece (Kriti)
*O. ehrenbergii*	Vulnerable	Lebanon
*O. libanoticum*	Vulnerable	Lebanon
*Salvia*		
*S. granatensis* (formerly *Rosmarinus tomentosus*)	Endangered	Spain (mainland)
*S. herbanica*	Critically Endangered	Spain (Canary Is.)
*S. peyronii*	Critically Endangered	Lebanon
*S. taraxacifolia*	Endangered	Morocco
*Sideritis*		
*S. cypria*	Vulnerable	Cyprus
*S. cystosiphon*	Critically Endangered	Spain (Canary Is.)
*S. discolor*	Critically Endangered	Spain (Canary Is.)
*S. gulendamii*	Endangered	Turkey
*S. infernalis*	Vulnerable	Spain (Canary Is.)
*S. javalambrensis*	Vulnerable	Spain (mainland)
*S. marmorea*	Critically Endangered	Spain (Canary Is.)
*S. scardica*	Near Threatened	Albania; Bulgaria; Greece (mainland); North Macedonia; Serbia; Turkey (Turkey-in-Europe)
*S. serrata*	Critically Endangered	Spain (mainland)
*S. reverchonii*	Endangered	Spain (mainland)
*S. veneris*	Critically Endangered	Cyprus
*Thymus*		
*T. albicans*	Vulnerable	Portugal (mainland); Spain (mainland)
*T. camphoratus*	Near Threatened	Portugal (mainland)
*T. capitellatus*	Near Threatened	Portugal (mainland)
*T. carnosus*	Near Threatened	Portugal (mainland); Spain (mainland)
*T. lotocephalus*	Near Threatened	Portugal (mainland)
*T. saturejoides*	Vulnerable	Algeria; Morocco

The information in the table was compiled from the IUCN Red List of Threatened Species. Version 2022-2. [https://www.iucnredlist.org (accessed on 20 December 2022)].

## Data Availability

Not applicable.
